# Influence of Dietary Compounds on Arsenic Metabolism and Toxicity. Part II—Human Studies

**DOI:** 10.3390/toxics9100259

**Published:** 2021-10-11

**Authors:** Monika Sijko, Lucyna Kozłowska

**Affiliations:** Department of Dietetics, Institute of Human Nutrition Sciences, Warsaw University of Life Sciences (SGGW-WULS), 159c Nowoursynowska Street, 02-776 Warsaw, Poland

**Keywords:** vitamins, minerals, inorganic arsenic, exposure, detoxification, metal toxicity, methylation

## Abstract

Exposure to various forms of arsenic (As), the source of which may be environmental as well as occupational exposure, is associated with many adverse health effects. Therefore, methods to reduce the adverse effects of As on the human body are being sought. Research in this area focuses, among other topics, on the dietary compounds that are involved in the metabolism of this element. Therefore, the aim of this review was to analyze the influence of methionine, betaine, choline, folic acid, vitamin B_2_, B_6_, B_12_ and zinc on the efficiency of inorganic As (iAs) metabolism and the reduction in the severity of the whole spectrum of disorders related to As exposure. In this review, which included 62 original papers (human studies) we present the current knowledge in the area. In human studies, these compounds (methionine, choline, folic acid, vitamin B_2_, B_6_, B_12_ and zinc) may increase iAs metabolism and reduce toxicity, whereas their deficiency may impair iAs metabolism and increase As toxicity. Taking into account the results of studies conducted in populations exposed to As, it is reasonable to carry out prophylactic activities. In particular nutritional education seems to be important and should be focused on informing people that an adequate intake of those dietary compounds potentially has a modulating effect on iAs metabolism, thus, reducing its adverse effects on the body.

## 1. Introduction

Exposure to arsenic (As) affects people living in many regions of the world. This problem occurs not only in less affluent areas (such as Chile, Argentina, Bangladesh, and Mexico), but also in several hotspot regions of Europe (Poland, Hungary, Serbia, Romania, Czech Republic, Croatia, Finland, Greece, and Italy) [1]. The source of exposure to different chemical forms of As can be both environmental and occupational. Environmental pollution can be of natural origin (rocks, soils, and volcanoes, among others) and can be caused by human activities (e.g., mining, burning fossil fuels, residues of agricultural chemicals in the soil and groundwater) [2,3]. Environmental sources also include water and food contamination. The As content in food products varies and depends on the type of product and its origin. Foods that contribute most to general population exposure to inorganic arsenic (iAs) include rice and rice-based products, grains and grain-based processed products, and drinking water [4]. The second source of As exposure is occupational exposure. As is used in many industries (agriculture; horticulture; mining; wood preservation; and production of, e.g., ammunition, glass, semiconductors, dyes) [2,3].

Both environmental and occupational exposures include exposure to inorganic as well as organic forms. Inorganic forms (such as As trioxide, As pentoxide, arsenous acid, and arsenic acid) are more toxic than organic forms (monomethylarsonic acid (MMA) and dimethylarsinic acid (DMA), arsenobetaine). Among iAs, As on the third and fifth oxidation state is the most toxic, with forms of the third oxidation state being more toxic [5,6,7].

Exposure to As can cause many adverse health effects. iAs has been classified as a carcinogenic compound [8]. Epidemiological studies in individuals exposed to As have shown an increased incidence of cancers including lung, kidney, liver, bladder, and skin cancers [9,10]. Long-term exposure to As in adults increased the risk of type 2 diabetes [11,12] and the risk of developing peripheral vascular disease [13], increased the incidence of skin lesions [14], and impaired lung function [15]. Furthermore, As exposure in children had a negative impact on cognitive abilities and induced neurological disorders [16,17].

The large number of people exposed to As and the many negative health effects caused by this exposure warrant, among other investigations, a deep analysis of the spectrum of health effects associated with As exposure and a search for ways that could reduce these adverse effects on the human body. Research in this area has focused, among other topics, on dietary compounds. There is no systematic summary of the results of human studies that have analyzed the effects of key dietary compounds on iAs metabolism and reduction in adverse effects caused by As exposure. Therefore, in this paper, we analyzed the results of studies in which the influence of methionine, choline, betaine, folic acid as donors of methyl groups and vitamins B_2_, B_6_, B_12_ and zinc as reaction cofactors in the aspect of efficiency of the metabolism process as well as reduction in the severity of the whole spectrum of disorders related to As exposure.

## 2. Methods

In this review, the electronic database PubMed was used. The following keywords were used to search for articles: arsenic and: methionine, betaine, choline, folic acid, folate, zinc, vitamin B, vitamin B_2_, vitamin B_6_, vitamin B_12_, riboflavin, pyridoxine, cobalamin. The review was based on the PRISMA statement for reporting systematic reviews and meta-analyses of studies that evaluate health care interventions: explanation and elaboration [18]. The search resulted in 2434 articles, excluding those unrelated to the topic of the study and those that examined the effects of complex plant extracts. Sixty-two (4 in vitro studies and 58 human studies) original peer-reviewed articles in English were included in the analysis, which studied the effects of methionine, betaine, choline, folic acid, vitamin B_2_, B_6_, B_12_, zinc on iAs metabolism and As-induced toxicity. Articles published between 1980 and 2020 were used, 95.2% of which were published after 2000.

## 3. Results

### 3.1. Folic Acid and Zinc as Modulators of iAs Metabolism and Toxicity—In Vitro Studies

The protective effect of folic acid (two studies) and zinc (two studies) has been analyzed in four studies on cell lines that were exposed to iAs. The results are summarized in Table 1.

#### 3.1.1. Folic Acid—iAs Metabolism

The association between folic acid and iAs metabolism was analyzed only in one in vitro study. In a study performed in Chang human hepatocytes, folate deficiency decreased the levels of methylated arsenicals, and supplementation with folic acid showing no effects on iAs metabolism [19].

#### 3.1.2. Folic Acid and Zinc—Toxicity of iAs

In one of the in vitro studies in Chang human hepatocyte folate supplementation decreased apoptosis, lipid peroxidation, and oxidative stress. Furthermore, folate deficiency enhanced the adverse effect of iAs, resulting in, inter alia, increased apoptosis, lipid peroxidation, and oxidative stress [19].

In the in vitro study performed on human embryonic kidney 293 cells, folic acid showed a protective effect against adverse effect of iAs by increasing the viability of cells and decrease the oxidative stress levels through decreasing the levels of reactive oxygen species and p66Shc expression [20].

Furthermore, a study with a human monocyte cell line, carried out in the presence of iAs, demonstrated that zinc deficiency increased oxidative stress (increased reactive oxygen species production) and inflammatory response (inter alia, through increased production of inflammatory markers) [21].

In the human keratinocyte cell lines exposed to iAs, zinc reduced DNA damage by affecting the production of poly(ADP-ribose) polymerase-1 (increased content zinc in this protein and its activity; decreased As binding with this protein) [22].

#### 3.1.3. Folic Acid and Zinc—Summary

In vitro studies have shown that folic acid deficiency decreased iAs metabolism and supplementation had no influence. Folic acid deficiency (1 h) could exacerbate the adverse effect of exposure to iAs in a short period of time, and the addition of folic acid (also only for 1 h) did not improve iAs metabolism; in both cases, a long exposure time to iAs (24 h) was used.

However, these compounds reduced the adverse changes induced by iAs in three cell lines, mainly through decreased apoptosis, oxidative stress and DNA damage. Although the studies were carried out on different cell lines and different doses of folic acid (10, 100 µM) zinc (2 µM) and iAs (2, 20 µM; 5 mM) with the same exposure times iAs (24 h), favorable results were obtained.

In two in vitro studies with iAs exposure (for 4 or 24 h), folic acid and zinc deficiency intensified oxidative stress, apoptosis and inflammation. In these cases, both short folic acid deficiency (1 h) and long zinc deficiency (4 weeks) had an adverse effect.

### 3.2. Relationship between Dietary Intake of Selected Compounds and iAs Metabolism and Toxicity

Twenty-two studies in subjects exposed to iAs (mainly in drinking water) analyzed the relationships between the intake of a whole range of nutrients and iAs metabolism as well as toxicity (Table 2).

#### 3.2.1. Nutrient Intake—iAs Metabolism

The main results of studies that analyzed the relationships between intakes of methionine, betaine, choline, vitamins B_2_, B_6_, B_12_, folate, zinc and biomarkers of iAs metabolism in children and then in adults are presented below.

The relationship between nutrient intake and iAs metabolism was reported in four studies conducted with children.

In the case of vitamin B_2_, no significant relationship was observed between its intake and the urinary excretion of As metabolites (%MMA, %DMA, and %iAs) [23]. A negative association between vitamin B_6_ intake and %MMA in urine was observed only in one study involving children, and there were no significant relationships between vitamin B_6_ intake and the percentage of DMA and iAs in the urine [23]. In contrast, in the study by Kurzius-Spencer et al. [24], no significant association was observed between vitamin B_6_ intake and the urinary excretion of iAs metabolites. Three studies showed no relationship between vitamin B_12_ intake and iAs metabolism in children [23,24,25]. Folate intake was negatively associated with urinary %MMA [25,26] and, additionally, in one of these studies, a positive association with urinary %DMA was observed [26]. In these studies, no association was observed between intake of folic acid and %iAs, tAs in the urine [25,26] and urinary excretion of iAs metabolites [24].

The relationship between nutrient intake and iAs metabolism was also analyzed in the adult population (10 studies).

In the study by Heck et al. [27], methionine intake (in groups of men and women) was positively associated with urinary %MMA, whereas no such relationship was observed in another study involving women [28]. In the study by Heck et al. [28], methionine intake also was positively associated with urinary %DMA [28], but in a subsequent study by these authors, this relationship was not significant [27]. Additionally, these two studies showed a negative association between methionine intake and %iAs in the urine [27,28]. Moreover, in the study by Heck et al. [29], a higher methionine intake was associated with an increased excretion of tAs in the urine. Another analyzed ingredient was betaine; there was no relationship between betaine intake and iAs metabolism [27,28].

Regarding choline, two studies showed intake of this compound was not associated with urinary %MMA, but a positive association was observed with the DMA/MMA ratio [27,28]. In contrast, one study demonstrated associations with the other iAs metabolites in urine (positive association of choline intake with %DMA and negative association with %iAs) [28].

The relationships between vitamin B_2_ intake and iAs metabolism have been analyzed in several studies. One study showed a positive association between vitamin B_2_ intake and urinary %MMA [27], while another study showed a negative association with this form [30]. Additionally, in several studies, there were no relationships [28,31,32,33]. In the study by Spratlen et al. [30], vitamin B_2_ intake was positively related with urinary %DMA, while no such relationship was shown in other studies [27,28,31,32,33]. In contrast, a negative association with %iAs was shown in the study by Spratlen et al. [30], while no such relationship was observed in other studies [27,28,31,32,33]. There was no significant relationship between vitamin B_2_ intake and urinary excretion of tAs [34]. One study revealed a positive association between vitamin B_2_ intake and first methylation step (MMA/iAs), and negative with the second methylation step (DMA/MMA) [27].

The relationship between vitamin B_6_ intake and iAs metabolism was analyzed in seven studies. In the study by Spratlen et al. [30], vitamin B_6_ intake was negatively associated with urinary %MMA and positively with %DMA. In the study by Kurzius-Spencer et al. [24], these relationships were not significant. Both studies showed a negative association with urinary %iAs [24,30], while the study by Argos et al. [34] showed a positive association with tAs in the urine. On the other hand, in several studies, there was no relationship between vitamin B_6_ intake and urinary %iAs metabolite excretion [27,28,31,33].

Vitamin B_12_ intake correlated positively with urinary %MMA in the study by Heck et al. [27]; other studies did not show such a relationship [24,28,30,32]. The study by Lopez-Carillo et al. [28] also showed a positive association with urinary %DMA, which was not observed in the studies of other authors [27,30,32]. Only in two studies, the intake of this vitamin was negatively associated with %iAs in the urine [27,28]. This relationship was not confirmed in other studies [24,30,32]. Positive associations with ratios of various forms of As in the urine were also observed in studies by Heck et al. [27] and Lopez-Carillo et al. [28]. However, the intake of this vitamin did not affect the urinary excretion of tAs [34].

None of the studies observed a relationship between folic acid intake and urinary %MMA and %DMA [27,28,30,31,32,33]. In contrast, the study by Howe et al. [35] showed that intake of sum of vitamins B_2_, B_6_, B_12_ and folate had a negative association with the proportion of monomethyl arsenic species. In turn, folate intake was negatively associated with urinary %iAs in one study [28], which was not shown in other studies [27,30,31,32,33]. Folate intake was positively associated with ratio DMA/iAs [28]. No relationship between folate intake and tAs in the urine was reported in the study by Argos et al. [34].

Associations between zinc intake and iAs metabolism were demonstrated in two studies. They included one negative association with urinary %MMA and a positive association with urinary %DMA [28,33]. Moreover, the study by Lopez Carillo et al. [28] demonstrated a negative association with urinary %iAs and a positive association with ratios DMA/MMA and DMA/iAs. 

#### 3.2.2. Nutrient Intake—Toxicity of As

Several studies have analyzed the relationship between nutrient intake and the severity of adverse changes in the body and the risk of developing diseases associated with exposure to As. 

In one study involving adults and children, an adverse effect of high vitamin B_6_ intake was observed; it was associated with an increased risk of diabetes and metabolic syndrome [31]. On the other hand, in another study, a low intake of vitamin B_2_, B_6_, B_12_ and folic acid in subjects exposed to iAs with drinking water was associated with worse cardiovascular outcomes (through increased pulse pressure and marginally systolic hypertension) [36]. A protective effect of nutrients was demonstrated in one study, in which a reduction in the severity of oxidative stress (reduced urinary 15-F_2t_-isoprostane) was observed in adults who had a higher intake of B vitamins [35].

However, no consistent effect was observed between folic acid intake and bladder cancer risk in adults [37]. Three studies demonstrated an increased risk of skin lesions in individuals who had low intakes of choline, vitamin B_2_, folate and zinc [38,39,40]. In turn, a reduced risk of As-related skin lesions was observed in adults who consumed higher amounts of vitamin B_2_, B_6_ and folic acid [41].

Low folic acid intake was not associated with cognitive performance in children, but higher intake was positively associated with cognitive abilities. In this study, moreover, several relationships were observed between tAs in the urine and cognitive performance depending on the level of folic acid intake, but it was an inconsistent effect [42]. In another study by the same author, there was no relationship between urinary tAs and achievement (broad math and reading scores) among children and B vitamin intake [43].

In turn, negative associations between vitamin B_12_ intake and tAs concentrations in toenails were observed in the study by Gruber et al. [44].

#### 3.2.3. Nutrient Intake—Summary

The results of human studies on the relationship between nutrient intake and iAs metabolism are inconclusive. They indicate that some nutrient intake may contribute to iAs elimination from the body. In several studies, the intake of such nutrients as methionine, choline, vitamin B_2_, vitamin B_6_, B_12_, folate, zinc was observed to be correlated with iAs metabolism. However, some of the correlations between nutrient intakes (betaine, choline, vitamin B_2_, vitamin B_6_, vitamin B_12_, and folate) and the urinary content of various forms of iAs suggest that these nutrients may or may not impair iAs metabolism. 

**Table 2:** Relationship between dietary compound intake and iAs metabolism and health effects related to As exposure.

Reference	Population	Dietary Assessment Methods	Component	Main Results
Desai et al., 2020 [23]	*n* = 290 Montevideo (Uruguay), children ~7 years	2 nonconsecutive 24 h recalls	vitamin B_6_—dietary intake	urine: %DMA (NS), %MMA (−), %iAs (NS)
			vitamin B_2_ and B_12_—dietary intake	urine: %DMA (NS), %MMA (NS), %iAs (NS)
Desai et al., 2020 [25]	*n* = 307 Montevideo (Uruguay), children ~7 years	2 nonconsecutive 24 h recalls	vitamin B_12_—dietary intake	urine: %DMA (NS), %MMA (NS), %iAs (NS)
			folate—dietary intake	urine: %DMA (NS), %MMA (−), %iAs (NS)
Kordas et al., 2016 [26]	*n* = 357 Montevideo (Uruguay), children ~5–8 years	2 nonconsecutive 24 h recalls	folate—dietary intake	urine: %DMA (+), %MMA (−), %iAs (NS), tAs (NS)
Kurzius-Spencer et al., 2017 [24]	*n* = 2420 U.S., adults and children > 6 years	24 h recall	vitamin B_6_—dietary intake	urine: %DMA (NS), %MMA (NS), %iAs (NS), DMA/MMA (NS) (in the group of children) urine: %DMA (NS), %MMA (NS), %iAs (−), DMA/MMA (NS) (in the group of adults)
			vitamin B_12_—dietary intake	urine: %DMA (NS), %MMA (NS), %iAs (NS), DMA/MMA (NS) (in the groups of adults and children)
			folate—dietary intake	urine: %DMA (NS), %MMA (NS), %iAs (NS), DMA/MMA (NS) (in the group of children) urine: %DMA (+), %MMA (NS), %iAs (−), DMA/MMA (NS) (in the group of adults)
Spratlen et al., 2018 [31]	*n* = 935 Arizona, Oklahoma, North Dakota, South Dakota, men and women >14 aged	FFQ	vitamin B_2_, vitamin B_6_, folic acid—dietary intake	urine: %DMAs (NS), %MMAs (NS), %iAs (NS)
			vitamin B_6_—dietary intake	risk for metabolic syndrome (+), risk for diabetes (+), HOMA2-IR (+)
			vitamin B_2_, folic acid—dietary intake	risk for metabolic syndrome (NS), risk for diabetes (NS), HOMA2-IR (NS)
Lopez-Carillo et al., 2016 [28]	*n* = 1027 Mexico, women	FFQ	methionine—dietary intake	urine: %DMA (+), %MMA (NS), %iAs (−), DMA/MMA (+), DMA/iAs (+)
			betaine—dietary intake	urine: %DMA (NS), %MMA (NS), %iAs (NS), MMA/iAs (NS), DMA/MMA (NS)
			choline—dietary intake	urine: %DMA (+), %MMA (NS), %iAs (−), DMA/MMA (+), DMA/iAs (+)
			vitamin B_2_, vitamin B_6_—dietary intake	urine: %DMA (NS), %MMA (NS), %iAs (NS), DMA/MMA (NS), DMA/iAs (NS)
			vitamin B_12_—dietary intake	urine: %DMA (+), %MMA (NS), %iAs (−), DMA/MMA (+), DMA/iAs (+)
			folate—dietary intake	urine: %DMA (NS), %MMA (NS), %iAs (−), DMA/MMA (NS), DMA/iAs (+)
			zinc—dietary intake	urine: %DMA (+), %MMA (−), %iAs (−), DMA/MMA (+), DMA/iAs (+)
Heck et al., 2009 [29]	*n* = 10,402 Bangladesh, men and women	FFQ	methionine—dietary intake	urine: tAs↑ and (+)
Heck et al., 2007 [27]	*n* = 1016 Bangladesh, men and women	FFQ	methionine—dietary intake	urine: %DMA (NS), %MMA (+), %iAs (−), MMA/iAs (+), DMA/MMA (NS)
			betaine—dietary intake	urine: %DMA (NS), %MMA (NS), %iAs (NS), MMA/iAs (NS), DMA/MMA (NS)
			choline—dietary intake	urine: %DMA (NS), %MMA (NS), %iAs (NS), MMA/iAs (NS), DMA/MMA (+)
			vitamin B_2_—dietary intake	urine: %DMA (NS), %MMA (+), %iAs (NS), MMA/iAs (+), DMA/MMA (−)
			vitamin B_6_—dietary intake	urine: %DMA (NS), %MMA (NS), %iAs (NS), MMA/iAs (NS), DMA/MMA (NS)
			vitamin B_12_—dietary intake	urine: %DMA (NS), %MMA (+), %iAs (−), MMA/iAs (+), DMA/MMA (NS)
			folate—dietary intake	urine: %DMA (NS), %MMA (NS), %iAs (NS), MMA/iAs (NS), DMA/MMA (NS)
Bommarito et al., 2019 [32]	*n* = 1166 Chihuahua (Mexico), men and women	FFQ	vitamin B_2_, vitamin B_12_, folate—sufficient and insufficient	urine: %DMAs↔, %MMAs↔, %iAs↔
Spratlen et al., 2017 [30]	*n* = 405 Arizona, Oklahoma, North Dakota, South Dakota, men and women	FFQ	vitamin B_2_, B_6_—dietary intake	urine: %DMA (+), %MMA (−), %iAs (−)
			vitamin B_12_, folate– dietary intake	urine: %DMA (NS), %MMA (NS), %iAs (NS)
Steinmaus et al., 2005 [33]	*n* = 87 U.S., men and women	HHHQ	zinc—dietary intake	urine: %DMA (+), %MMA (−), %iAs (NS)
			vitamin B_2_, vitamin B_6_, folate—dietary intake	urine: %DMA (NS), %MMA (NS), %iAs (NS)
Argos et al., 2010 [34]	*n* = 9833 Araihazar (Bangladesh), men and women	FFQ	vitamin B_6_—dietary intake	urine: tAs (+)
			vitamin B_2_, vitamin B_12_, folate—dietary intake	urine: tAs (NS)
Chen et al., 2007 [36]	*n* = 10,910 Bangladesh, men and women	FFQ	vitamin B_2_, B_6_, B_12_, folate—low intake level	ORs of high pulse pressure↑, ORs of systolic hypertension↑ (weak association)
Howe et al., 2017 [35]	*n* = 418 New Hampshire, men and women	FFQ	vitamin B_2_, vitamin B_6_, vitamin B_12_, folate—sum of B vitamin—dietary intake	urine: proportion of MMAs (−), 15-F_2t_-IsoP (−)
Koutros et al., 2018 [37]	*n* = 2366 Maine, New Hampshire, Vermont, case with bladder cancer and control group	DHQ	folate—high and low intake level	ORs of risk of bladder cancer↔ (weak association)
Melkonian et al., 2012 [38]	*n* = 16,391 Araihazar (Bangladesh), cases with skin lesions and control group	FFQ	vitamin B_2_, folate—low intake level	keratotic skin lesion risk (+)
Deb et al., 2012 [39]	*n* = 208 West Bengal, cases with skin lesions and control group	24 h recall	choline, vitamin B_2_, zinc—low intake level	ORs of skin lesions↑ (in the group of women)
			choline, zinc—low intake level	ORs of skin lesions↑ (in the group of men)
			vitamin B_6_, vitamin B_12_, folate	ORs of skin lesions↔ (in the group of women and men)
Mitra et al., 2004 [40]	*n* = 384 West Bengal (India), cases with skin lesions and control group	24 h recall	folate—low intake level	ORs of skin lesions↑
			vitamin B_2_, vitamin B_6_, zinc	ORs of skin lesions↔
Zablotska et al., 2008 [41]	*n* = 10,628 Araihazar (Bangladesh), men and women	FFQ	vitamin B_2_, B_6_, folic acid—high intake level	PORs risk for skin lesions↓
			vitamin B_12_	PORs risk for skin lesions↔
Desai et al., 2018 [42]	*n* = 328 Montevideo (Uruguay), children ~5–8 years	2 nonconsecutive 24 h recalls	folate—low intake level	cognitive performance (NS) tAs—concept formation (−), tAs—scores of numbers reversed subtest (+), tAs—cognitive efficiency (+)
			folate—mean intake level	scores on: verbal comprehension (+), visual auditory learning (+), verbal ability (+), general intellectual abilities (+) tAs—sound integration scores (+)
			folate—high intake level	scores on: visual auditory learning (−), concept formation (+), numbers reversed (+), cognitive efficiency (+) tAs—concept formation (+)
Desai et al., 2020 [43]	*n* = 239 Montevideo (Uruguay), children ~5–8 years	2 nonconsecutive 24 h recalls	vitamin B_2_, vitamin B_6_, vitamin B_12_, folate—dietary intake	broad math and reading scores (calculation, math facts fluency, applied problems, sentence reading fluency, letter word identification, passage comprehension) and urinary tAs (NS)
Gruber et al., 2012 [44]	*n* = 920 New Hampshire, men and women	FFQ	vitamin B_12_—dietary intake	toenail: tAs (−)

↑—significant increase; ↓—significant decrease; ↔—no significant changes; (+)—positively association; (−)—negatively association; (NS)—no significant association; DHQ—diet history questionnaire; DMA—dimethylarsinic acid; DMAs—monomethyl arsenic species; FFQ—food frequency questionnaire; HHHQ—health habits and history questionnaire; HOMA2-IR—Homeostatic Model Assessment Index 2; iAs—inorganic arsenic; MMA—monomethylarsonic acid; MMAs—monomethyl arsenic species; ORs—odds ratios; PORs—prevalence odds ratios; tAs—total arsenic species; 15-F_2t_-IsoP—15-F_2t_-isoprostane.

The authors also observed a relationship between a high intake of B vitamins and folic acid and a reduction in the adverse changes associated with exposure to iAs (decreased oxidative stress, reduced risk of As-related skin lesions, and increased cognitive abilities). In turn, low intake (choline, B vitamins, folic acid, and zinc) was associated with the deterioration of cardiovascular outcomes and an increased risk of skin lesions. The differences in these results may be due to the levels of nutrient intake (higher intake was connected with a reduction in the negative effects associated with iAs exposure, while a low intake may exacerbate these effects).

However, in several studies, there are conflicting data that require clarification. No consistent effect was observed between B vitamins and folic acid intake, and risk of diabetes and bladder cancer, as well as cognitive performance and achievements in children. In these studies, the author suggested that the lack of, or unclear, effect may be due to the low variability in vitamin intake, or that intake was above a sufficient level in the majority of participants. Additionally, the results could also be influenced by additional metabolic differences (e.g., genetic variation or differences in iAs metabolism—the study was conducted on children, adults and patients with bladder cancer). In one of these studies, a high intake of vitamin B_6_ was associated with diabetes-related outcomes, but this effect was unclear because, in most studies, low consumption contributed to the severity of the negative effects and, therefore, this aspect requires further analysis.

### 3.3. Folic Acid and Zinc Supplementation

Table 3 includes results from studies in which folic acid (eight studies) and zinc (one study) were supplemented in a population exposed to As.

#### 3.3.1. Folic Acid and Zinc Supplementation—iAs Metabolism

Five studies analyzed the protective effect of folic acid and zinc supplementation on iAs metabolism. Changes in iAs metabolite concentrations (inter alia, decreased concentrations of MMA in the blood and increased urinary excretion of DMA) were observed in people taking folic acid supplementation [45]. In a study by Bozack et al. [46], folic acid supplementation in subjects with low and normal blood concentrations of this compound also altered iAs metabolism profile (by increased percentage dimethyl-arsenical species and decreased %iAs and monmethylarsenical species in the urine). In the study by Peters et al. [47] conducted on a Bangladeshi population, folic acid supplementation at a higher dose (800 µg/db) reduced the concentration of tAs in the blood. This effect persisted even after 12 weeks from the end of supplementation, but no such effect was observed with a lower dose of this vitamin (400 µg/db). Folic acid supplementation in participants with betaine concentrations below the median affected iAs metabolism (inter alia, through an increased percentage of dimethyl-arsenical species and decreased percentage of monomethyl arsenical species in the urine) [48]. In contrast, in a group of children, zinc supplementation decreased urinary %DMA, but did not affect the concentration of other As biomarkers in the urine [49]. In a group of Bangladeshi residents exposed to iAs in drinking water supplementation with folic acid, there was an influence on parameters related to iAs metabolism through increased plasma choline and betaine concentration and a percentage decrease in dimethylglycine in the plasma. There were no significant differences between groups receiving lower (400 µg/day) and higher (800 µg/day) doses of folic acid [50].

**Table 3:** Results of studies conducted in people with As exposure and supplementation with folic acid or zinc.

Reference	Research Model	Study Description	Main Results
Gamble et al., 2007 [45]	Bangladesh, adults	CG (*n* = 62)—placebo (orally, for 12 weeks) G1 (N = 68)—folic acid 400 µg/day (orally, for 12 weeks)	G1 vs. CG blood: MMA↓, tAs↓, DMA↔ urine: DMA↑ (after 1 week) DMA↔(after 12 week)
Bozack et al., 2019 [46]	Bangladesh, adults	CG (*n* = 90)—placebo (orally, for 12 weeks) G1 (*n* = 133)—folic acid 400 µg/day (orally, for 12 weeks) G2 (*n* = 129)—folic acid 800 µg/day (orally, for 12 weeks)	G1, G2 vs. CG plasma: folate↑, homocysteine↓ RBC folate↑ urine: %iAs↓, %MMAs↓, %DMAs↑
			G2 vs. G1 urine: %MMAs↓
		G1 (*n* = 68)—folic acid 400 µg/day (orally, for 12 weeks) and after that placebo (orally, for 12 weeks) G2 (*n* = 60)—folic acid 800 µg/day (orally, for 12 weeks) and after that placebo (orally, for 12 weeks) G1a (*n* = 65)—folic acid 400 µg/day (orally, for 24 weeks) G2a (*n* = 69)—folic acid 800 µg/day (orally, for 24 weeks)	G1a, G2a vs. G1, G2 urine: %iAs↓, %MMAs↓, %DMAs↑
Peters et al., 2015 [47]	Bangladesh, adults	CG (*n* = 102)—placebo (orally, for 12 weeks) G1 (*n* = 153)—folic acid 400 µg/day (orally, for 12 weeks) G2 (*n* = 151)—folic acid 800 µg/day (orally, for 12 weeks)	G1 vs. CG plasma folate↑, RBC folate↑, geometric mean of blood tAs↑, percentage decline in geometric mean blood tAs from baseline↓
			G2 vs. CG plasma folate↑, RBC folate↑, geometric mean of blood tAs↓, percentage decline in geometric mean blood tAs from baseline↑
		CG (*n* = 102)—placebo (orally, for 24 weeks) G1 (*n* = 76)—folic acid 400 µg/day (orally, for 12 weeks) and after that placebo (orally, for 12 weeks) G2 (*n* = 74)—folic acid 800 µg/day (orally, for 12 weeks) and after that placebo (orally, for 12 weeks) G1a (*n* = 77)—folic acid 400 µg/day (orally, for 24 weeks) G2a (*n* = 77)—folic acid 800 µg/day (orally, for 24 weeks)	G2, G2a vs. CG geometric mean of blood tAs↓
			G1a vs. G1 geometric mean of blood tAs↔, percentage decline in geometric mean of urinary and blood tAs↔
			G2a vs. G2 geometric mean of blood tAs↔, percentage decline in geometric mean of urinary and blood tAs↔
Bozack et al., 2020 [48]	Bangladesh, adults	CG (*n* = 104)—placebo (orally, for 12 weeks) G1 (*n* = 156)—folic acid 400 µg/day (orally, for 12 weeks) G2 (*n* = 154)—folic acid 800 µg/day (orally, for 12 weeks)	G1 vs. CG participants with betaine concentrations below the median: urine: decreases in ln(%iAs)↑, decrease in %MMAs↑, increases in %DMAs↑
			G2 vs. CG participants with betaine concentrations below the median: urine: decreases in ln(%iAs)↑, increases in %DMAs↑
Kordas et al., 2017 [49]	Mexico, children (6–7 years)	CG (*n* = 151)—placebo (orally, for 6 months) G1 (*n* = 144)—zinc oxide 30 mg/day (orally, for 6 months)	G1 vs. CG urine: %DMA↓, %MMA↔, %iAs↔, tAs↔
Hall et al., 2016 [50]	Bagladesh, adults	CG (*n* = 101)—placebo (participants received arsenic-removal water filters and had been drinking water from wells with water As concentration >50 µg/L at least 3 years) G1 (*n* = 152)—folic acid 400 µg/day (orally, for 12 weeks) and after that placebo (participants received arsenic-removal water filters and had been drinking water from wells with water As concentration >50 µg/L at least 3 years) G2 (*n* = 149)—folic acid 800 µg/day (orally, for 12 weeks) and after that placebo (participants received arsenic-removal water filters and had been drinking water from wells with water As concentration >50 µg/L at least 3 years)	G1, G2 vs. CG plasma: choline↑, betaine↑, percentage decrease in DMG↑
			G1 vs. G2 plasma: choline↔, betaine↔, percentage decrease in DMG↔
Dani, 2019 [51]	Women (16 year-old)—chronic arsenic intoxication	*ex juvantibus* therapy: torasemide 10–20 mg/day, thiamine 300 mg/day, magnesium 5 mg/day, folic acid 5 mg/day and metamizole and simeticon (on-demand)	nuchal scalp hair shafts: As—undetectable morning urine: As—undetectable afternoon urine: As 50 nmol/L symptoms (leg cramps, abdominal pains)↓
Howe et al., 2017 [52]	Bangladesh, adults	CG (*n* = 104)—placebo (orally, for 12 weeks) G1 (*n* = 156)—folic acid 400 µg/day (orally, for 12 weeks)	G1 vs. CG blood: PTHMs↔
Ghose et al., 2014 [53]	India, patients with symptoms of arsenic toxicity	CG (*n* = 45)—drinking arsenic free water (orally, for 6 months) G1 (*n* = 32)—folic acid 5 mg/day (orally, for 6 months)	G1 vs. CG skin score↓, systemic disease score↑ (overall: clinical symptoms of arsenicosis↓)

↑—significant increase; ↓—significant decrease; ↔—no significant changes; CG—control group; DMA—dimethylarsinic acid; DMAs—dimethyl arsenical species; DMG—dimethylglycine; G1—group 1; G2—group 2; iAs—inorganic arsenic; MMA—monomethylarsonic acid; MMAs—monomethyl arsenical species; PTHMs—post-translational histone modifications; RBC—red blood cell; tAs—total arsenic species.

#### 3.3.2. Folic Acid Supplementation—Toxicity of As

In one study, symptoms of chronic arsenic intoxication were analyzed in an adolescent girl who was taking globules (containing iAs). Treatment with thiamine and folic acid, among others, reduced the adverse symptoms and the concentration of iAs in urine and hair [51]. In contrast, folic acid supplementation did not alter post-translational histone modifications in peripheral blood mononuclear cells from Bangladeshi adults [52]. Ghose et al. [53] showed that folic acid supplementation reduced adverse symptoms in patients with chronic As toxicity.

#### 3.3.3. Folic Acid and Zinc Supplementation—Summary

In four studies, folic acid supplementation (in doses 400 and 800 µg/day for 12 or 24 weeks) improved iAs metabolism in adults (mainly through increased urinary DMA concentration and decreased tAs concentration in the urine and the blood). In one of these studies, only a higher dose of folic acid improved iAs metabolism. In turn, zinc supplementation (30 mg/day for 6 months) in children resulted in a different effect (decreased urinary DMA concentration).

In two studies, folic acid supplementation (5 mg/day) reduced the adverse symptoms associated with As poisoning, while in one study, folic acid supplementation with a small dose (400 µg/day) had no effect on epigenetic regulation. A short period of supplementation (12 weeks) with folic acid or using a low dose of folic acid (400 mg/day) could make this effect invisible. The authors also indicate that the As-removing water filter was used during the study, which could counteract the effects caused by folic acid supplementation.

### 3.4. Blood and Tissues Nutrients Concentration

Table 4 presents a summary of the results of 29 human studies in which the relationship between the concentrations of dietary compounds (in blood and other tissue) and As metabolism was analyzed, as well as severity of adverse health effects associated with As exposure.

#### 3.4.1. Blood Nutrient Concentration—iAs Metabolism

Eight studies analyzed the relationship between blood micronutrient concentrations and urinary As metabolites in children. No relationship was observed between plasma vitamin B_6_ levels and urinary excretion of As metabolites (%MMA, %DMA, and %iAs) [24]. The results of three studies [24,25,54] did not demonstrate the relationship between vitamin B_12_ concentration in the plasma/serum and As metabolites in the urine. In turn, the results of two studies demonstrated an increase in the %DMA and a decrease in the %MMA, %iAs in the urine of the group of children with high concentrations of vitamin B_12_ and folate in their plasma [55] in the second study showed a positive association between serum vitamin B_12_ concentration and urinary concentrations of DMA [56]. Additionally, results of three studies showed no relationship between the concentration of plasma/serum folate and urinary %MMA [24,54,57]. Plasma/serum folate concentration was positively associated with the percentage and concentration of DMA in the urine [56,57,58] and negatively with urinary %iAs [54,57]. In contrast, in the study by Kurzius-Spencer et al. [24], folate concentration in the serum had no relationship with urinary %DMA and %iAs, and in the study by Desai et al. [25], no relationship was observed with any urinary iAs metabolites.

Family iAs metabolism was only analyzed in one study. A strong pattern of metabolism was shown between siblings. Additionally, in siblings, positive correlations between urinary iAs/methylated As, monomethylarsonate/dimethylarsinate a methionine, vitamin B_6_, B_12_ folate in the blood were observed. These correlations between parents and children were much lower [59].

The relationship between nutritional status and iAs metabolism has also been analyzed in the adult population (12 studies).

One study found that plasma vitamin B_6_ concentration was not associated with the urinary excretion of As metabolites (%MMA, %DMA, and %iAs) [24].

A lower %MMA and higher %DMA in the urine were observed in adults with a low concentration of vitamin B_12_ in the plasma compared to individuals with a higher concentration of this vitamin. The study also noted that the concentration of vitamin B_12_ in the plasma was positively associated with urinary %MMA, but inversely associated with urinary %iAs. Such a relationship was stronger in the group of subjects with an adequate folate concentration in the plasma [60]. The study by Zhu et al. [56] demonstrated that serum vitamin B_12_ concentration was positively associated with urinary concentrations of DMA. However, the results of other studies showed no relationship between plasma and serum vitamin B_12_ concentration and urinary As metabolites [24,61].

In contrast, three studies found no relationship between plasma/serum vitamin B_12_ concentrations and urinary excretion of As metabolites (%MMA, %DMA, and %iAs) in pregnant women [62,63,64]. Only the study by Laine et al. [62] demonstrated a negative correlation between maternal serum vitamin B_12_ with tAs in the urine. Moreover, in women, the concentration of vitamin B_12_ in the plasma was inversely associated with arsenate [65] and positively associated with %iAs [62] in cord blood.

In one study, serum/plasma folate concentration was positively associated with urinary %MMA [58], while in the study by Gamble et al. [61], it was negatively associated with urinary %MMA. Two studies demonstrated a positive association between folate concentration in the serum/plasma and urinary %DMA [56,61]. In turn, the results of another study did not report such a relationship [58]. Moreover, in the study by Gamble et al. [61] plasma folate concentration was negatively associated with urinary %iAs. In turn, in the study by Kurzius-Spencer et al. [24] no relationship was shown between serum folate levels and urinary iAs metabolites.

In a Bangladeshi population, individuals with low plasma folate concentrations showed a correlation between decreased ratio of glutathione-to-glutathione disulfide and higher blood levels of tAs, as well as having increased %MMA and decreased %DMA in the urine [66].

The study by Chung et al. [67] analyzed the relationship between gene polymorphisms, iAs metabolism and plasma folate. Abnormal iAs metabolism and decreased plasma folate levels were observed in patients with urothelial carcinoma. Subjects with the 5,10-methylenetetrahydrofolate reductase CT or TT genotype had lower percentage dimethylarsenic acid in the urine and a lower folate concentration in the plasma than those with the CC genotype. A positive correlation was also observed between plasma folate concentration and percentage urinary dimethylarsenic acid in the control group.

In Bangladeshi women, folate concentration in the plasma/serum was inversely associated with arsenate in the maternal blood [65], and in pregnant women negatively correlated with percentage monomethyl arsenicals in the cord serum [62]. In contrast, three studies did not demonstrate significant correlations between plasma folate levels and urinary As metabolites in pregnant women [62,63,64]. Only in women with both higher As exposure level and plasma folate concentration was a reduced iAs level in the urine noted [63].

Two studies reported no significant relationship between plasma zinc concentrations and urinary As metabolites in pregnant women [62,64]. Only in subjects with the highest exposure level were higher plasma zinc concentrations associated with increased %MMA and %iAs, and decreased %DMA [63].

#### 3.4.2. Blood and Tissue Nutrient Concentration—Toxicity of As

Many studies have focused on analyzing the relationship between nutritional status and As concentrations in various tissues and adverse health effects in individuals exposed to As.

The relationship between As exposure, nutritional status, and DNA methylation was analyzed in three studies. In elderly men, no association was observed between the plasma concentrations of vitamin B_6_ and B_12_ and DNA methylation. However, in men with a low concentration of folate in the plasma, a positive association with one repetitive element (increased Alu DNA methylation) was observed, whereas in men with a higher concentration of plasma folate, the effect was the opposite [68]. Similarly, the study in Bangladeshi adults with high plasma folate concentration (above 9 nmol/L) showed negative correlation between urinary or plasma tAs and methylation of peripheral blood leukocyte DNA (lower [3H]-methyl incorporation) [69]. In turn, in women with folate deficiency in the plasma, an inversely association was observed between the tAs concentration in toenail and total histone 3 levels in the plasma [70].

Howe et al. [52] demonstrated positive associations between the plasma concentration of vitamin B_12_ (among women) and choline (among men) and global levels of post-translational histone modifications.

Several studies have reported the relationship between blood concentrations of nutrients and the risk of disease development or the severity of adverse body changes associated with an exposure to As.

A higher odds ratio of type 1 diabetes by the percentage of monomethylates As was observed in individuals with a higher concentration of plasma folate (stronger relationship between percentage monomethylated As and type 1 diabetes). No significant relationship was shown for type 2 diabetes between percentage monomethylates As and plasma folate or vitamin B_12_ concentration [71]. It was observed in the study by Chung et al. [72] that low a concentration of folate in the plasma, global 5-methyl-2′-deoxycytidine levels, and a high concentration of total urinary As levels was related to an increased risk of urothelial carcinoma. The study conducted in a West Bengal population, did not demonstrate any relationship between the concentration of folate, methionine, vitamin B_6_, vitamin B_12_ in the blood and the susceptibility to develop skin lesions [73]. Subjects with low plasma folate concentrations had an increased risk of skin lesions, whereas no such relationship was observed in subjects with lower vitamin B_12_ concentrations [74].

Low concentrations of folate and vitamin B_12_ in the plasma were indirectly (through association with decreased iAs metabolism) related to an increased childhood developmental delay (increase in odds ratio of developmental delay) [55]. In the study by Desai et al. [43], higher broad math scores were observed in subjects with higher vitamin B_12_ in the serum and tAs concentration in the urine.

In subjects exposed to As, zinc and tAs concentrations in different tissues were analyzed. In pregnant women living in Wuhan (the largest industrial city), a positive correlation between tAs and zinc concentration in blood was observed [75]. On the other hand, in patients with blackfoot disease, decreased zinc concentration in the hair, urine, blood as well as increased tAs concentration in hair and urine were observed [76,77,78]. On the other hand, in groups of workers, a higher concentration of tAs in the lung tissue and in the blood was observed, but no decreased concentration of zinc in these tissues was shown [79,80]. Moreover, in the study by Tutkun et al. [80], the correlation between Zn and tAs was negative and in the workers group higher levels of inflammatory markers (interleukins 6, 10 and tumor necrosis factor-α) was also observed, but no correlation was observed between these cytokines and Zn.

**Table 4:** Relationship between concentration of dietary compound in blood and other tissues and iAs metabolism and health effects related to As exposure.

Reference	Population	Measure of Component Status	Main Results
Hall et al., 2009 [54]	*n* = 165 Bangladesh, children (6 years old)	plasma: vitamin B_12_	urine: %DMA (NS), %MMA (NS), %iAs (NS)
		plasma: folate	urine: %DMA (NS), %MMA (NS), %iAs (−)
Skroder Loveborn et al., 2016 [57]	*n* = 488 Bangladesh, children (9 years old)	plasma: folate	urine: %DMA (+), %MMA (NS), %iAs (−)
Lin et al., 2019 [55]	*n* = 266 Taiwan, children (preschool aged)	plasma: vitamin B_12_ and folate	urine: %DMA↑, %MMA↓, %iAs↓ (in the group with high concentrations vitamin B_12_ and folate)
			ORs of development delay↑ (in the group with low concentrations vitamin B_12_ and folate)
Desai et al., 2020 [25]	*n* = 307 Montevideo (Uruguay), children ~7 years	serum: vitamin B_12_, folate	urine: %DMA (NS), %MMA (NS), %iAs (NS)
Zhang et al., 2019 [58]	*n* = 11,016 US, adults and children (≤18 years)	serum: folate	urine: %DMA (+), MMA (NS) (in the group of children) urine: %DMA (NS), %MMA (+) (in the group of adults)
Zhu et al., 2018 [56]	*n* = 3099 U.S., adults and children (6–19 years)	serum: vitamin B_12_, folate	urine: DMA (+) (in the group of children and in the group of adults)
Kurzius-Spencer et al., 2017 [24]	*n* = 2420 U.S., adults and children >6 years	plasma: vitamin B_6_ serum: vitamin B_12_, folate	urine: %DMA (NS), %MMA (NS), %iAs (NS), DMA/MMA (NS) (in the groups of adults and children)
Chung et al., 2002 [59]	*n* = 44 Chile, adults and children (6–14 years)	blood: methionine, vitamin B_6_, vitamin B_12_, folate	urine: iAs/methylated As (+), MMA/DMA (+)
Hall et al., 2009 [60]	*n* = 778 Bangladesh, adults	plasma: vitamin B_12_	urine: %DMA↑, %MMA↓, %iAs↔ (in the vitamin B_12_ deficient group compared to vitamin B_12_ sufficient group) urine: %DMA (NS), %MMA (+), %iAs (−)
Gamble et al., 2005 [61]	*n* = 300 Bangladesh, adults	plasma: vitamin B_12_	urine: %DMA (NS), %MMA (NS), %iAs (NS)
		plasma: folate	urine: %DMA (+), %MMA (−), %iAs (−)
Niedzwiecki et al., 2014 [66]	*n* = 376 Bangladesh, adults	plasma: folate	plasma: GSH/GSSG ratio association with urine: %DMA (−), %MMA (+), SMI (+), blood: tAs (−) (in the folate deficient group)
Chung et al., 2010 [67]	*n* = 450 cases with urothelial carcinoma and control group	plasma: folate	urine: %DMA↓, %MMA↑, %iAs↑, tAs↑ (cases with urothelial carcinoma) urine: %DMA↓ (controls with 5,10-methylenetetrahydrofolate reductase CT or TT genotype) urine: %DMA (+) (in the control group)
Hall et al., 2007 [65]	*n* = 30 pairs Bangladesh, women and children (newborn)	plasma: vitamin B_12_	cord blood: percentage arsenate (−) (in the group of women)
		plasma: folate	blood: percentage arsenate (−) (in the group of women)
Laine et al., 2018 [62]	*n* = 197 Mexico, women (pregnant)	serum: vitamin B_12_	urine: %DMA (NS), %MMA (NS), %iAs (NS), tAs (−) cord serum: %iAs (+)
		serum: folate	urine: %DMA (NS), %MMA (NS), %iAs (NS), tAs (NS) cord serum: %MMAs (−)
Li et al., 2008 [63]	*n* = 753 Bangladesh, women (pregnant)	plasma: vitamin B_12_, folate, Zn—high and low values	urine: %DMA↔, %MMA↔, %iAs↔ (in the group at the low As exposure level)
		plasma: folate—high values	urine: %iAs↓ (in the group at the highest As exposure level)
		plasma: Zn—high values	urine: %DMA↓, %MMA↑, %iAs↑, SMI↓ (in the group at the highest As exposure level)
Gardner et al., 2011 [64]	*n* = 324 Bangladesh, women (pregnant)	plasma: vitamin B_12_, folate, Zn	urine: %DMA (NS), %MMA (NS), %iAs (NS)
Lambrou et al., 2012 [68]	*n* = 581 Boston, men (elderly)	plasma: vitamin B_6_, vitamin B_12_	blood: Alu (NS), Long Interspersed Nucleotide Element-1 (NS)
		plasma: folate	blood: Alu (+), Long Interspersed Nucleotide Element-1 (NS) (in the low folate group)
			blood: Alu (−), Long Interspersed Nucleotide Element-1 (NS) (in the high folate group)
Pilsner et al., 2007 [69]	*n* = 294 Bangladesh, adults	plasma: folate	[^3^H]-methyl incorporation association with tAs in the urine, plasma (−) (in the high folate group)
Tauheed et al., 2017 [70]	*n* = 85 Bangladesh, women	plasma: folate	tAs concentration in toenail association with plasma total H3 (−) (in the folate deficient group)
Howe et al., 2017 [52]	*n* = 324 Bangladesh, adults	plasma: choline	peripheral blood mononuclear cells: H3K36me2 (+) (in the men group)
		plasma: vitamin B_12_	peripheral blood mononuclear cells: H3K79me2 (+) (in the women group)
		plasma: folate	peripheral blood mononuclear cells: H3K36me2 (NS), H3K36me3 (NS), H3K79me2 (NS) (in the men and women group)
Grau-Perez et al., 2017 [71]	*n* = 688 U.S., adults and children	plasma: vitamin B_12_	ORs of type 1 and type 2 diabetes by %monomethylated As↔
		plasma: folate—high values	ORs of type 1 diabetes by %monomethylated As↑ ORs of type 2 diabetes by %monomethylated As↔
Chung et al., 2019 [72]	*n* = 534 Taiwan, cases with urothelial carcinoma and control group	plasma: folate	ORs of urothelial carcinoma↑ (low folate level and global 5-MedC, high tAs in the urine)
Chung et al., 2006 [73]	*n* = 372 West Bengal, cases with skin lesions and control group	blood: methionine, vitamin B_6_, vitamin B_12_, folate	ORs of skin lesions↔
Pilsner et al., 2009 [74]	*n* = 548 Bangladesh, cases with skin lesions and control group	plasma: vitamin B_12_—low values	ORs for development skin lesions↔
		plasma: folate—low values	ORs for development skin lesions↑
Desai et al., 2020 [43]	*n* = 239 Montevideo (Uruguay), children ~5–8 years	serum: vitamin B_12_	urine: tAs and broad math score (+)
Gong et al., 2020 [75]	*n* = 406 Wuhan, women (pregnant) and control group (non-pregnant)	blood: Zn	blood: tAs (+) (in the group of pregnant women)
Wang et al., 1994 [76]	*n* = 218 Taiwan, cases with blackfoot disease and control group	hair: Zn	hair: Zn↓, tAs↑ (in the group of patients with blackfoot disease)
Tsai et al., 2004 [77]	*n* = 136 Taiwan, cases with blackfoot disease and control group	urine: Zn	urine: Zn↓, tAs↑ (in the group of patients with blackfoot disease)
Lin and Yang, 1988 [78]	*n* = 56 cases with blackfoot disease and control group	blood, serum, urine: Zn	blood, serum, urine: Zn↓ (in the group of patients with blackfoot disease) urine: tAs↔(in the group of patients with blackfoot disease) hair: tAs↑ (in the group of patients with blackfoot disease)
Gerhardsson and Nordberg, 1993 [79]	*n* = 110 smelter workers and control group (from urban and rural area)	lung tissue: Zn	lung tissue: Zn↔, tAs↑
Tutkun et al., 2019 [80]	*n* = 135 Ankara, men—workers (exposed to As) and control group	serum: Zn	blood: tAs↑, Zn↔, IL-6↑, IL-10↑, TNF-α↑ (in the workers group) correlation tAs—Zn (−) (in the workers group)

↑—significant increase; ↓—significant decrease; ↔—no significant changes; (+)—positively association; (−)—negatively association; (NS)—no significant association; As—arsenic; DMA—dimethylarsinic acid; GSH/GSSG—glutathione/glutathione disulfide; H3—histone 3; H3K36me2—histone 3 lysine 36 dimethylation; H3K36me3—histone 3 lysine 36 trimethylation; H3K79me2—histone 3 lysine 79 dimethylation; iAs—inorganic arsenic; IL-6—Interleukin 6; IL-10—interleukin 10; MMA—monomethylarsonic acid; MMA/DMA—monomethylarsonate/dimethylarsinate; MMAs—monomethyl arsenical species; ORs—odds ratios; SMI—secondary methylation index; tAs—total arsenic species; TNF-α—tumor necrosis factor-α; Zn—zinc; 5-MedC—5-methyl-2′deoxycytidine.

#### 3.4.3. Concentration of Nutrients in Blood and Other Tissues—Summary

The results of human studies on the relationship between the blood concentrations of dietary compounds and iAs metabolism are also inconclusive. They indicate that the concentration of dietary compounds in the blood may be related to iAs metabolism and contribute to its elimination from the body. This is indicated by several studies in which it was observed that the blood concentration of vitamin B_12_ and folate correlated with iAs metabolism. However, some of the correlations between the blood concentration of these nutrients (folate, B_6_, B_12_, and zinc) and the urinary content of various forms of iAs suggest that these nutrients may or may not impair iAs methylation.

Three studies showed that nutritional status (choline, vitamin B_12_, folate—higher plasma/serum concentrations) may be related to a decrease in adverse health effects (affected methylation DNA, histone modification, and children achievement) in individuals exposed to As. The results of this study are in line with expectations, since the appropriate status of these nutrients can alleviate the adverse health effects associated with exposure to As. Numerous studies also observed a relationship between concentration of these nutrients (folate—low and higher plasma concentration; vitamin B_12_—low plasma concentration) and increased risk of disease and adverse body changes associated with an exposure to As (increased DNA methylation, risk of type 1 diabetes and urothelial carcinoma, skin lesions, and developmental delay). Folate deficiency (five studies) and vitamin B12 deficiency (one study) enhanced adverse effects of iAs exposure, and only in one study higher concentration of folate exacerbated them. These results indicate that both a deficiency and an excess of these compounds may contribute to enhancing the toxicity of As. The level of exposure to iAs does not appear to be a differentiating factor, as the adverse effects associated with folic acid deficiency occurred in populations with low (including elderly men, preschool children, and women) and high exposure (cases with skin lesions and urothelial carcinoma). In several studies, no relationships (or the relationship was unclear) between blood/plasma concentration of methionine, vitamins B_6_, B_12_, folate a DNA methylation, risk of type 2 diabetes and skin lesions were found. Moreover, in pregnant women, patients with blackfoot disease and workers exposed to As, elevated tAs concentrations in many tissues were connected with disturbances in zinc concentrations.

## 4. Conclusions

Many studies have also focused on analyzing the relationship between dietary compounds (intake, supplementation mainly folic acid, and blood concentrations), and iAs metabolism, as well as exposure-related disorders. The intake and blood concentrations of certain dietary compounds (methionine, choline, vitamin B_2_, B_6_, B_12_, folic acid, and zinc) showed a relationship with an improvement in iAs metabolism and were associated with reduction in adverse health effects. It was also shown that not only the deficiency (folate), but also the excess of some dietary compounds (vitamin B_12_, folic acid, zinc) may impair iAs metabolism and may increase adverse health effects. Human studies, to date, are inconclusive, because many factors influenced the results. This signals the need for a more detailed analysis of the relationship between the nutrient intake of involved in iAs metabolism, nutritional status as well as the severity and source of iAs exposure. Considering the promising results of the studies conducted so far, it seems reasonable that individuals exposed to iAs should consume natural products rich in methionine, choline, folic acid, B vitamins (B_2_, B_6_, B_12_) and zinc. Products rich in methionine (meat: turkey, beef, pork and milk, tofu, and Brazil nuts), choline (eggs, beans, and broccoli; meat: chicken, pork, and beef), folic acid (green leafy vegetables), B-group vitamins (meat, eggs, dairy product, leafy greens, and legumes), and zinc (meat, nuts, and cereal products). Nutrition education focusing on an adequate dietary intake of methionine, choline, zinc, folic acid, and B vitamins (B_2_, B_6_, and B_12_)—nutrients which potentially have modulating effects in iAs metabolism and toxicity—should be used in prevention efforts for populations exposed to iAs.

## Figures and Tables

**Table 1:** Results of in vitro studies with iAs exposure and folic acid or zinc treatment.

Reference	Research Model	Study Description	Main Results
Xu et al., 2010 [19]	Chang human hepatocytes	CG—normal folate medium 2.3 µM (1 h) and sodium arsenite 20 µM (for 24 h) G1—folate-deficient medium (1 h) and sodium arsenite 20 µM (for 24 h) G2—folate-supplemented medium 10 µM (1 h) and sodium arsenite 20 µM (for 24 h)	G1 vs. CG intracellular tAs↔, methylated arsenicals↓ viability↓, early apoptosis↑, late apoptosis↑, caspase-3 cleavage↑, PARP cleavage↑, percentage of cells with collapsed mitochondrial membrane potential↑, ROS↑, TBARS↔, GSH↓, CAT↓, SOD↔ cytochrome c: in mitochondria↓, in cytosol↑
			G2 vs. CG intracellular tAs↔, methylated arsenicals↔ viability↑, early apoptosis↔, late apoptosis↔, caspase-3 cleavage↓, PARP cleavage↓, percentage of cells with collapsed mitochondrial membrane potential↓, ROS↓, TBARS↓, GSH↑, CAT↔, SOD↔ cytochrome c: in mitochondria↑, in cytosol↓
Ma et al., 2015 [20]	HEK293ET cells (human embryonic kidney 293 cells)	CG—sodium arsenite 5 mM G1—folic acid 100 µM and sodium arsenite 5 mM	G1 vs. CG cell viability↑, mRNA level of GDF1↑, ROS↓, expression of p66Shc↓
Wong et al., 2019 [21]	THP-1 (human monocyte cell line)	CG—zinc sulfate 4 µM (for 4 weeks) and after that sodium arsenite 10 µM (for 24 h) G1—zinc sulfate 0 µM (for 4 weeks) and after that sodium arsenite 10 µM (for 24 h)	G1 vs. CG Zn total and intracellular↓, ROS↑
		CG—zinc sulfate 4 µM (for 4 weeks) and after that sodium arsenite 10 µM (for 4 h) G1—zinc sulfate 0 µM (for 4 weeks) and after that sodium arsenite 10 µM (for 4 h)	G1 vs. CG HO-1↑, SOD↔, CAT↔
		CG—zinc sulfate 4 µM (for 4 weeks) and after that sodium arsenite 1 µM (for 4 h) G1—zinc sulfate 0 µM (for 4 weeks) and after that sodium arsenite 1 µM (for 4 h)	G1 vs. CG intracellular zinc↓, transcript levels of: ICAM1↑, IL6↑, CXCL8↑
Sun et al., 2014 [22]	HaCaT cells (human keratinocyte cell line)	CG—sodium arsenite 2 µM (for 24 h) G1—zinc chloride 2 µM (for 24 h) and sodium arsenite 2 µM (for 24 h)	G1 vs. CG zinc content in PARP-1↑, PARP-1 activity↑

↑—significant increase; ↓—significant decrease; ↔—no significant changes; CAT—catalase; CG—control group; CXCL8—C-X-C motif chemokine ligand 8; G1—group 1; GDF 1—growth differentiation factor 1; GSH—glutathione; HO-1—*heme oxygenase*-*1*; ICAM1—intercellular adhesion molecule 1; IL-6—Interleukin 6; PARP—poly(ADP) polymerase; PARP-1—poly(ADP) polymerase-1; ROS—reactive oxygen species; SOD—superoxide dismutase; tAs—total arsenic species; TBARS—thiobarbituric acid reactive substances.

## Data Availability

Not applicable.
